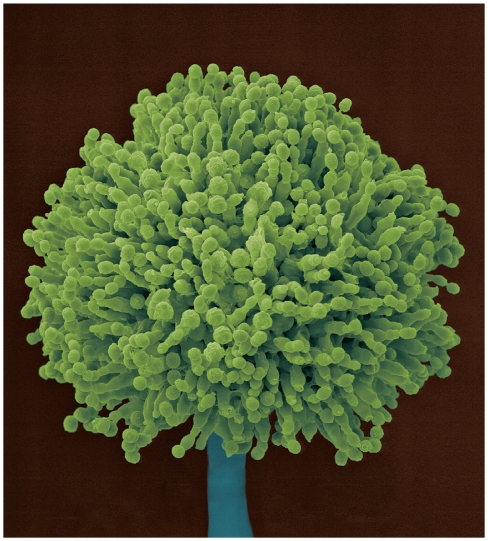# Carcinogenic Crops: Analyzing the Effect of Aflatoxin on Global Liver Cancer Rates

**DOI:** 10.1289/ehp.118-a258a

**Published:** 2010-06

**Authors:** Tanya Tillett

**Affiliations:** **Tanya Tillett**, MA, of Durham, NC, is a staff writer/editor for *EHP*. She has been on the *EHP* staff since 2000 and has represented the journal at national and international conferences

Tree nuts and groundnuts, along with maize and other grains, can harbor aflatoxins, naturally occurring fungal metabolites that have been identified as risk factors for developing liver cancer. This association has most often been seen in people infected with hepatitis B virus (HBV). A new study examines the af latoxin/HBVrelationship to offer the first quantitative risk assessment of the number of liver cancer cases worldwide caused by aflatoxin **[*EHP*** **118:818–824; Liu and Wu]**.

Although a relatively rare malignancy in developed countries, liver cancer is a common health threat in developing regions of the world including Southeast Asia, China, and sub-Saharan Africa. These same regions have higher prevalence of HBV infection as well as higher levels of aflatoxin contamination in food due to a lack of resources to control the fungi *Aspergillus flavus* and *Aspergillus parasiticus*, which infiltrate crops and produce aflatoxin. Research has shown that individuals with chronic HBV infection and aflatoxin exposure are up to 30 times more at risk for liver cancer than uninfected individuals exposed to aflatoxin.

In the current study, researchers analyzed information on food consumption patterns, aflatoxin biomarker levels in serum and urine, HBV prevalence, and population size in different world regions to quantify the subsequent risk of developing liver cancer. The investigators found that consumption of maize and ground-nuts was higher overall in African and Asian countries than in wealthier, more developed nations, leading to increased aflatoxin exposure. However, risk of aflatoxin-induced liver cancer could vary widely within a given nation: urban populations with more diverse diets had lower aflatoxin exposures than their rural counterparts, and there also was a lower HBV prevalence in urban populations.

The authors concluded that uncontrolled exposure to aflatoxin may cause 4.6–28.2% of all liver cancer cases globally, with China, Southeast Asia, and sub-Saharan Africa bearing the brunt of the burden. This broad range reflects the uncertainty and variability of the available data on aflatoxin exposure and HBV prevalence. One thing does seem certain, they write: if more interventions to control aflatoxin and its health risks (for instance, improved storage protocols and vaccination for HBV) were administered in regions where they are most needed, liver cancer incidence could be significantly reduced worldwide.

## Figures and Tables

**Figure:**